# Are physical fitness outcomes in patients attending cardiac rehabilitation determined by the mode of delivery?

**DOI:** 10.1136/openhrt-2018-000822

**Published:** 2018-07-16

**Authors:** Alexander S Harrison, Lars Tang, Patrick Doherty

**Affiliations:** 1 Department of Health Sciences, University of York, York, UK; 2 National Knowledge Centre for Rehabilitation and Palliative Care, University of Southern Denmark and Odense University Hospital, Copenhagen, Denmark

**Keywords:** exercise, cardiac rehabilitation, physical health, secondary prevention

## Abstract

**Background:**

Cardiac rehabilitation (CR) is a well-evidenced and effective secondary intervention proven to reduce mortality and readmission in patients with cardiovascular disease. Improving physical fitness outcomes is a key target for CR programmes, with supervised group-based exercise dominating the mode of the delivery. However, the method of traditional supervised CR fails to attract many patients and may not be the only way of improving physical fitness.

**Methods:**

Using real-world routine clinical data from the National Audit of Cardiac Rehabilitation across a 5-year period, this study evaluates the extent of association between physical fitness outcomes, incremental shuttle walk and 6 min walk test, and mode of delivery, delivered as traditional supervised versus facilitated self-delivered.

**Results:**

The proportion of patients receiving each mode were 80.6% supervised with 19.4% to self-delivered. The study analysis comprised of 10 142 patients who were included in the two models. The self-delivered group contained a greater proportion of females and older patients. The regression model showed no clinical or statistical significance between mode of delivery and post-CR physical fitness outcomes.

**Conclusions:**

This study is unique as it has identified through a routine clinical population that regardless of the mode of delivery of rehabilitation, patients improve their physical fitness outcomes at meaningful levels. This study provides a strong evidence base for patients to be offered greater choice in the mode of CR delivery as improvements in physical fitness are comparable.

**Key questions**

**What is already known about this subject?**

- Cardiac rehabilitation (CR) is a well-evidenced healthcare intervention that is successfully delivered to over 60 000 people in the UK each year. The intervention should be patient tailored and its mode of delivery varies from supervised to self-delivered. The evidence around the differences in outcome from these modes of delivery is lacking and has yet to look at physical fitness outcomes in routine populations. It has been shown in previous work that there is no statistical difference in patients’ fitness, although this was in trial populations that are often not representative of routine populations. Additionally, for other outcomes such as psychosocial well-being in routine populations there was also no difference between modes. This will be the first study to investigate physical fitness outcomes, such as incremental shuttle walk test and 6-min walk test, with the mode of delivery.

**What does this study add?**

- This study adds to the growing evidence base of mode of delivery and its lack of association with patient’s outcomes. For a long time, CR has thought to only be delivered in supervised gym-based sessions; however, with this study we know that not only are the outcomes of patients comparable but also that both groups of patients on average are exceeding meaningful clinical differences throughout their duration of the programme.

**How might this impact on clinical practice?**

- In the UK, CR is still predominantly delivered as supervised group-based rehabilitation, with it making up ~80% of the delivery type. Additionally, only 40% of programmes in 2017 record as using self-delivered CR. This study, along with others, that highlight the lack of differences in outcome for patients attending either type of rehabilitation can provide a strong evidence base for patients to be offered greater choice in the mode of CR delivery as improvements in physical fitness are comparable.

## Introduction

Cardiac rehabilitation (CR) is a well-evidenced intervention that remains effective in the modern era of cardiology.^1 2^ The aims of CR are to address and change lifestyle risk factors and promote physical fitness and mental health.^3 4^

The evidence for CR is from experimental and observational studies and shows that CR is effective at reducing mortality, both all-cause and cardiac along with readmissions.^1 2^ However, the majority of this evidence is based on traditional supervised group-based CR as opposed to facilitated primarily self-delivered modes of delivery. In 2017, Cochrane reviewed randomised controlled trial evidence for the differences between home-based and group-based rehabilitation in terms of health-related quality of life, exercise capacity and readmissions.^5^ They found that in all outcomes there was no significant association between the mode of delivery and where the patients were post-CR. Historically, researchers and healthcare funders have separated rehabilitation into home-based and group-based; however, recently the literature has also considered the level of supervision to be an important factor in terms of the delivery of rehabilitation.^6 7^

Although there is growing evidence in the trial populations that mode of delivery is not significantly associated with a range of outcomes, there is in parallel an acknowledgement that trials may not be representative of routine populations. Notwithstanding the known benefits of Cochrane reviews of CR, there are concerns about the populations being representative of routine care (eg, average age of patients within the trials (56 years, range 48–70 years) and women accounting for less than 15% of the population).^1^ In the most recent National Audit of Cardiac Rehabilitation (NACR) annual report, women made up 30% and the average age was 67 which is substantially older than the trials.^8^ Moreover, the intervention within the trials may not contain the variety or nuances that are present in real-life/routine care. Due to the differences in population and potential intervention, it is important to address questions around association between mode and outcomes in routine populations as well as in trials.

The UK NACR showed that in 2016, 80% of rehabilitation was delivered as group-based, with other methods such as home-based, web-based and telephone making up the other 20%.^8^ According to the British Association for Cardiovascular Rehabilitation and Prevention (BACPR) core components, the mode of delivery should be menu-based, with interventions centred on patients’ needs and preferences.^3 4^ The lack of choice in the CR offer shows that programmes are underusing modes of delivery proven to be effective at reducing risk factors and promoting lifestyle change.^5^ In fact, many programmes in the UK still only offer group-based CR with ~60% of programmes not offering any form of self-delivered (home-based, web-based or telephone-based) rehabilitation to any patients.^8^ The UK, Europe and the USA continue to aspire to challenging uptake ambitions in the region of 65% to 70%.^9 10^ Recent findings from clinical data and clinical review identify a lack of choice in the menu of routine practice CR and make recommendations for more options appealing to patients’ preferences and meeting their needs all of which will help overcome traditionally barriers to participation in CR such as older, female and non-native language speaking patients.^8 11^

British and European guidelines and core components suggest that CR is best delivered by a multidisciplinary team (MDT), through a variety of modes of delivery.^1 2^ A study based in Denmark, using the CopenHeart data, found that patients assigned to supervised group-based or self-delivered home-based found no difference in their perceived exertion levels postintervention nor exercise effects.^6 7^ A recent study conducted using data from the NACR showed that across the two delivery types, supervised versus self-delivery, there was no significant association with psychosocial health outcomes.^12^ This provides the context for an emerging hypothesis testing the likelihood that physical fitness outcomes do not differ between the delivery type and that patients can benefit from either approach.

This study aimed to assess whether the mode of delivery, as supervised or self-delivered, is associated with improved physical fitness outcomes as measured through the 6 min walk test (6MWT) and the incremental shuttle walk test (ISWT).

## Methods

This study was reported according to the Strengthening the Reporting of Observational Studies in Epidemiology (STROBE) guidelines.^13^

### Data

The study used data from a routinely collected audit of CR, the NACR. The NACR collects data from CR programmes across the UK and has a 74% coverage through online data entry.^8^ The electronic data come from 224 programmes, which collect data on patient’s demographics, baseline risk factors and characteristics, the type of CR received and outcomes derived from pre-CR and post-CR assessment. Along with patient level characteristics, the audit also collects service level factors such as the number of patients seen (volume), staffing hours and the extent of staff in their MDT.

Patients were included if they had an initiating event between 1 April 2012 and 31 March 2017. The initiating event was the diagnosis or treatment that deemed the patient eligible for CR. All patient groups, except heart failure, were included in the main analysis, such as myocardial infarction (MI), percutaneous coronary intervention (PCI) and coronary artery bypass.^14 15^Patients with a primary diagnosis of heart failure were not included as this group were only recently added to the NACR dataset and at present there is insufficient sample for inclusion. To be included, patients needed to have (1) a completed CR and (2) a recorded mode of delivery. To account for reporting bias, the population without a recorded mode of delivery were compared for baseline demographics such as age and gender.

### Mode of delivery

The NACR records the routine delivery CR in the UK, which includes core rehabilitation consisting of exercise sessions, education sessions and lifestyle advice as guided by the BACPR core components. The exercise sessions are supervised/facilitated by trained competent professionals to maximise patient benefit.^3^ The modes of delivery recorded in the NACR, includes both supervised and self-delivered levels.^8^ For this study, mode of delivery for each patient was coded from NACR variables, including group-based, home-based and web-based, into supervised (with staff present) and facilitated self-delivered (with contact but staff not required for the exercise component). Patients recorded as receiving delivery classified as ‘other’ were excluded from the study due to the lack of descriptive information; this equalled 3% of patients, and these were assessed for differences in demographics to test the extent by which our final sample was representative. Other factors about the service were included as covariates.

### Outcome measures

The study, accounting for baseline assessment scores, explored predictors of post-CR outcomes for the ISWT and 6MWT expressed in metres walked.^16–18^ The study used patients’ final score which is collected on average 9 weeks after the initial baseline assessment.^8^ The analysis also included the baseline walking score for all patients, to accurately account for their walking ability prior starting rehabilitation.

### Statistical analysis

The analyses were conducted in STATA 13.1. Baseline characteristics were compared across groups using χ^2^for categorical variables or t-test for continuous variables.^19^ Regression models were built to investigate whether, accounting for covariates, the supervised and self-delivered methods for mode of delivery were associated with outcomes post-CR.

Relevant important covariates were included in the analysis, where they were evidenced in the literature or significant in preliminary analysis. Age (years), gender (male/female), number of comorbidities and employment status have been shown to influence the outcomes following a variety of different rehabilitation interventions, including CR.^20–22^ Employment status was coded as employed/retired or unemployed, and this is because previous research found that employed and retired states have similar effects on outcomes.^20^ The duration of CR (length of CR) was also included in the analysis along with staffing profile, total staff hours, MDT and total centre volume after being evidenced in previous research.^20 21^ The duration was calculated from the start to the end of core rehabilitation, which is advised by the BACPR to be at least 8 weeks.^3^ Due to heterogeneity in the completion of sessions, the study was unable to include sessions and thus intensity of the intervention as a covariate. The staffing information comes from an annual survey, performed routinely by the NACR to gain centre level information such as staff profile, hours and funding type. Because the mode of delivery was a patient-level variable, it was important to take into account the relative size and staffing profile of the centre where the patient received the CR.

Hierarchical linear regressions were used to account for different levels of patient and centre level data as part of the investigation of association between mode of delivery, as an independent variable, and physical fitness outcomes as the dependent variable. Statistical level for significance was p<0.05. Data model checking was performed to ensure that the models were a good fit through assumptions associated with the regressions.

## Results

### Study population

**Figure 1:**
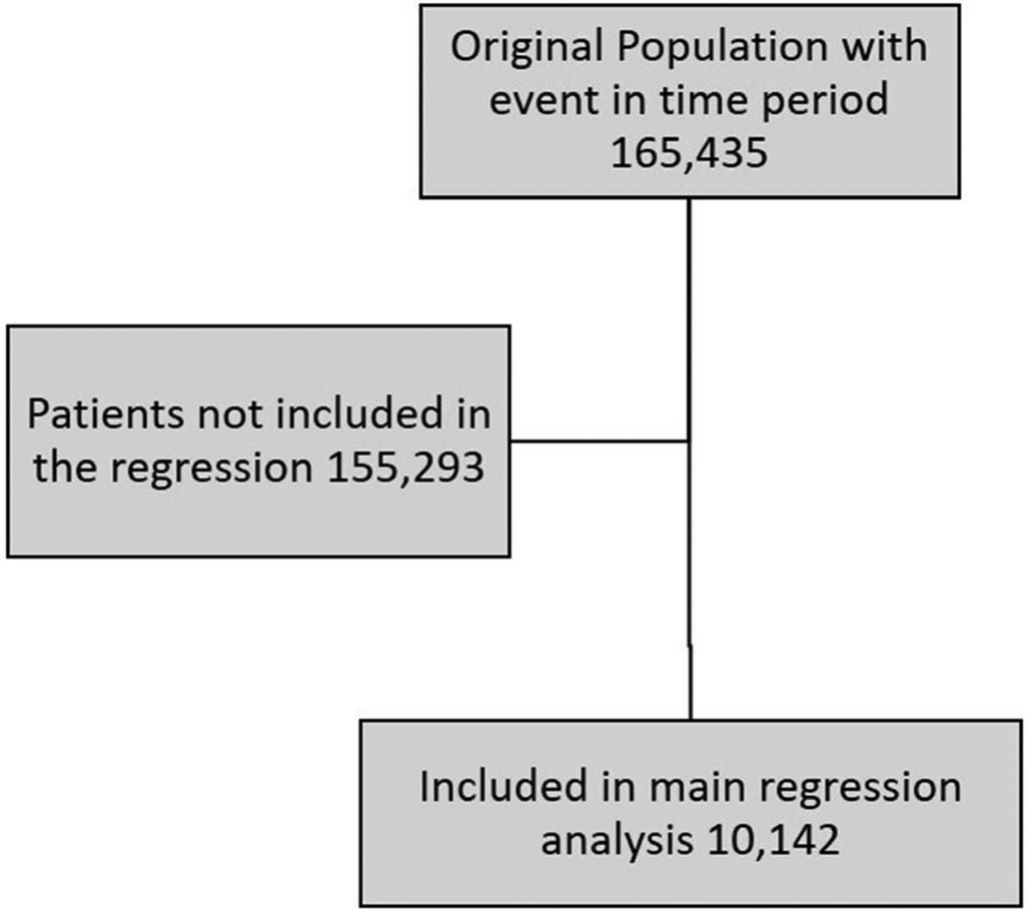
Flow diagram of the population included in the study based on having their event within April 2012–March 2017, having a recorded mode of delivery and completing cardiac rehabilitation. The population included in the analysis was compared with the full original population and were not significantly different.

The overall study population comprised 1 65 435 patients from the full dataset with an initiating event within the time period. The flow diagram in figure 1 shows the total population and those included in the regressions models. The diagnosis/treatment split was 78.9% conventional CR population (MI 12.6%, MI+PCI 31.6%, PCI 18.1% and coronary artery bypass grafting (CABG) 16.7%) and the remainder ‘Other’, such as angina.

The mode of delivery distribution, seen in table 1, was similar to that of the wider CR population and that seen in the annual statistics report, with 80.6% receiving supervised and 19.4% in the self-delivered group. The proportion of females was lower in the supervised mode, which was significant (p<0.001). The self-delivered group also contained older, more employed, previous partnered and patients with ‘other’ treatments than the conventional PCI and CABG. These differences were all significant. Additionally, the length of CR in the self-delivered group was on average 10 days longer with a total mean duration of 73 days. Each mode of delivery population were deemed similar and representative of routine care when compared for age, gender and other demographics.

**Table 1:** Patient characteristics across the two modes of delivery: supervised and self-delivered cardiac rehabilitation

	Supervised		Self-delivered		Total		Pearson χ^2^ value
	Count	%	Count	%	Count	%	
No of patients (%)	133 386	80.6	32 049	19.4	165 435		
Gender							
Female	33 172	25.7	9474	30.7	42 646	26.6	321.4 (<0.001)
Body measurement							
>30 BMI	27 906	30.9	5439	30.8	33 345	30.9	0.075 (0.784)
Employment status							
Employed	67 765	84	12 850	78	80 615	83	373.5 (<0.001)
Marital status							
Partner	73 412	78.4	15 743	75.3	89 155	77.8	110.9 (<0.001)
Previous partner	12 377	13.2	3314	15.9	15 691	13.7	
Cardiac treatment							
PCI	65 098	48.8	14 721	45.9	79 819	48.2	534.3 (<0.001)
CABG	19 726	14.8	3750	11.7	23 476	14.2	
Other treatment	32 048	24.0	9465	29.5	41 513	25.1	

	Mean	SD	Mean	SD	Mean	SD	Mean difference
Mean age (years)	65	12	67	12	65	12	2.2 (<0.001)
Total no of comorbidities	1.70	1.69	1.56	1.72	1.67	1.70	0.14 (<0.001)
Core rehabilitation duration start to end including assessment (days)	69.70	43.21	89.53	59.33	73.25	47.13	19.8 (<0.001)

BMI, body mass index; CABG; coronary artery bypass grafting; PCI, percutaneous coronary intervention.

The two-population’s physical baseline scores were also compared (table 2). The supervised group had, for both physical fitness measures, higher baseline scores by 30 m for 6MWT and 24 m for ISWT. The difference was also statistically significant (p<0.001).

**Table 2:** Baseline patients’ physical outcome scores across the two modes of delivery: supervised and self-delivered

	Supervised		Self-delivered		Total		Mean difference (p values)
	Mean (SD)	Count	Mean (SD)	Count	Mean (SD)	Count	
Six minute walk test metres at assessment 1	332.8 (132.8)	12 708	302.9 (134)	1440	329.7 (133)	14 148	29.9 (<0.001)
Shuttle walk test metres at assessment 1	356.9 (176)	19 137	332.8 (201)	2644	354.0 (179)	21 781	24.1 (<0.001)

Table 3 shows the extent of change post-CR. At a first level of analysis, not accounting for covariates, the supervised group’s ISWT change was statistically significantly higher in comparison to the self-delivered group, with a mean difference of 12.9 (p<0.001). However, the change seen for the 6MWT was greater in the self-delivered group, this was a 7 m greater change in the self-delivered group (p=0.007). Overall, the differences between the modes were not of clinical significance and patients attending either mode had meaningful clinical difference changes post - CR for this population.

**Table 3:** Change in patients physical outcomes’ post-cardiac rehabilitation across the two modes of delivery, supervised and self-delivered

	Supervised			Self-delivered			Total			Mean difference (p values)
	Mean (SD)	% change from baseline	Count	Mean (SD)	% change from baseline	Count	Mean (SD)	% change from baseline	Count	
Six minute walk test metres change	64.3 (65.8)	19.3	7215	57.4 (57.9)	19	732	63.7 (65.2)	19.3	7947	-6.9 (0.007)
Shuttle walk test metres change	102.7 (117.4)	28.8	11 133	115.6 (139.1)	34.7	1486	104.2 (120.2)	29.4	12 619	12.9 (<0.001)

### Outcomes

The regression model (table 4) showed that there was no significant difference between the mode of delivery and the post-CR physical fitness outcomes for either measure (p>0.05). The inclusions of predictors such as age, gender, baseline physical fitness score and service quality were justified and were statistically significant. The models had an R^2^ of 69%–85% and met the assumptions of uniform variance and linearity. The final population included in the regression model were compared with the wider study and routine care population and were deemed to be representative in terms of age, gender and other covariates. The full regression models for each outcome are included as online supplementary material which includes all covariates and the model descriptive.

10.1136/openhrt-2018-000822.supp1 Supplementary data

**Table 4:** Results from the hierarchical logistic regression analysis; association between mode of delivery and physical fitness outcomes post-CR

	Coefficient	Significance	95% CI	Snijders/Bosker R^2^	Observations
Six minute walk test metres at assessment 2	−1.38	0.806	−12.383 to 0.778	0.846	3653
Shuttle walk test metres at assessment 2	0.31	0.957	−11.111 to 0.690	0.690	6175

## Discussion

This study set out to investigate whether patients attending supervised or self-delivered CR had different outcomes, in terms of physical fitness. The study’s main analysis found that there was no significant difference in patient’s physical fitness outcomes and the mode of delivery they received, either supervised or self-delivered. This is the first study of routine CR patients to investigate physical fitness outcomes and the mode of delivery. This has been shown in trial populations to have similar relationship, Cochrane in a 2017 review found no association between home-based/group-based rehabilitation for patients post-CR exercise capacity.^5^ Additionally, this conclusion builds on other research, investigating delivery mode and psychosocial health outcomes.^12^ The combination of routine CR evidence and trial evidence results in a strong case for patients to have a menu-based approach offering supervised and facilitated self-delivered rehabilitation options.

The overall study population consisting of 1 65 435 patients and the regression population (n = 10 142), were representative of modern UK CR. The patient population in the analysis was checked against the population with no mode of delivery reported; the valid population were deemed as not significantly different in age, gender and baseline physical fitness measures. The age, gender and comorbidity demographics were similar to the national level data.^8^

This population had a high level of female participation. The total proportion of female was 26.6%, which is comparable with the overall NACR population (30%) and much higher than those recruited into the trials in the Cochrane 2017 review, where some studies had no female participants.^5^ Additionally, our study looked at mode defined as supervised and facilitated self-delivered. The self-delivered modes included not only home-based as per Cochrane but also structured and facilitated web and e manual based approach which is increasingly being provided as an option in routine practice.

The population taking up the self-delivered mode is older, includes more females and a greater proportion of other cardiology treatments. Across the world, there are well-evidenced barriers to CR entry in females and older patients.^23–26^ The current uptake for CR in the UK is 51%, which, although one of the top levels across the globe, falls short of targets such 65% set by NHS England. To meet these uptake targets and make CR more available to all eligible patients, greater utilisation of other modes, such as self-delivered should be considered. Having a menu-based approach, with the offer of CR being inclusive of more than just group-based, is essential for maximising patient participation.^11^ This study indicates that two traditionally under-represented patient groups (females and older patients) attend self-delivered mode of delivery in greater proportions; wider adoption of this approach will reduce such inequalities and potentially increase uptake generally.

The change in physical fitness from baseline is for all modes, larger than the meaningful clinical difference.^17 18^ This highlights that attending CR, through either mode, leads to a meaningful improvement in physical fitness for patients.

One possible reason for the lack of adoption for self-delivered rehabilitation is perhaps due to worry of safety surrounding non-supervised CR. This has been studied, and in 2014 a trial investigated the use of high-intensity interval training in CR patients.^27^ Although this was in a younger trial population, the results found that home-based non-supervised group do comparably well. Additionally, the training in both settings was deemed safe.^27^

### Limitations

One limitation that the study experienced was that although exercise testing is essential for setting objectives and assessing risk, the number of patients with pre-CR and post-CR physical fitness measurements was low. In 2016, NACR reported that less than one-third of patients had recorded physical fitness measurements either ISWT or 6MWT. This does limit the study results in that there may have been some reporting bias. However, the included population was verified against the wider eligible population in terms of demographics and characteristics, so the authors are confident in the regression model.

Another limitation with this study is that the study could not include intensity/dose of rehabilitation. The length of rehabilitation was included as a covariate as duration; however, the NACR currently has insufficient information regarding the number of sessions to calculate the dose. Although session data have just commenced as part of NACR data collection and will be available for further studies in 2019.

This study excluded patients with heart failure due to their difference in expected walking ability to the wider CR population such as re-vascularised patients. This strengthens our study as it reduces heterogeneity of our study population and additionally justifies future work into this subpopulation.

## Future work

This study’s results show that either mode is beneficial for physical fitness. A finding in the 2017 Cochrane review was that the adherence rate was greater in home-based CR.^5^ This study did not compare adherence rates between supervised and self-delivered CR. Future work will investigate whether the evidence shown in trials, in terms of adherence, is also true in routine CR.

## Conclusion

This study finds, for the first time in a routine clinical population, that physical fitness post-CR improves to a clinically meaningful level independent of the mode of delivery. The population taking part in self-delivered CR is higher in proportion of female and older patients. With CR continuing to fail to appeal to many eligible patients, adopting a more menu-based approach which uses modes such as self-delivered is likely to reduce such inequalities in access to CR. The regression model which accounted for patient demographics and service level factors showed no difference, clinical or statistical between mode and post-CR outcomes. This is the first study to investigate the association between mode and physical fitness in routine patient populations. The results show that the population receiving self-delivered benefit as much as supervised group supporting the equivalence of these modes of delivery.
