# Supplementary material for: Are physical fitness outcomes in patients attending cardiac rehabilitation determined by the mode of delivery?
Source: Open Heart. 2018 Jul 16;5(2):e000822. doi: 10.1136/openhrt-2018-000822 (PMC6059269; doi:10.1136/openhrt-2018-000822)
Supplement: Supplementary data [file openhrt-2018-000822supp001.pdf]

Appendices

Table 1. Results from the hierarchical logistic regression analysis; association between mode of delivery and Six min walk test metres post CR

	Coef.	Sig.	95% CI	
**Baseline Six min walk (metres)**	0.802	<0.001	0.780	0.824
**Gender (Female)**	-10.189	<0.001	-14.923	-5.455
**BMI (>30)**	-11.575	<0.001	-15.891	-7.258
**Employment (Unemployed)**	-8.256	0.014	-14.820	-1.691
**Employment (Retired)**	-8.082	0.009	-14.167	-1.997
**Marital Status(Partner)**	5.288	0.200	-2.806	13.381
**Marital Status (Previous Partner)**	5.657	0.245	-3.888	15.203
**Baseline Anxiety (Anxious)**	-0.201	0.953	-6.899	6.497
**Baseline Depression (Depressed)**	-4.984	0.237	-13.246	3.279
**Cardiac Treatment (PCI)**	-4.121	0.250	-11.148	2.905
**Cardiac Treatment (CABG)**	0.769	0.855	-7.492	9.029
**Cardiac Treatment (Other)**	-9.331	0.015	-16.829	-1.834
**Age (years)**	-1.131	<0.001	-1.405	-0.858
**Total Comorbidities**	-1.780	0.003	-2.963	-0.596
**Mode of Delivery (Self-delivered)**	-1.380	0.806	-12.383	9.623
**Staff Hours (hours)**	-0.015	0.447	-0.052	0.023
**MDT Team (>=3 staff types)**	-2.099	0.651	-11.181	6.983
**Volume (number of patients)**	-0.032	0.065	-0.066	0.002
**Constant**	231.307	<0.001	205.855	256.758
R^2 = 0.846, included sample size 3,653				

Table 2. Results from the hierarchical logistic regression analysis; association between mode of delivery and Shuttle walk test metres post CR

	Coef.	Sig.	95% CI	
**Baseline Six min walk (metres)**	0.796087	<0.001	0.776888	0.815286
**Gender (Female)**	-35.3601	<0.001	-42.1118	-28.6084
**BMI (>30)**	-33.3737	<0.001	-39.3009	-27.4464
**Employment (Unemployed)**	-20.3505	<0.001	-29.3347	-11.3662
**Employment (Retired)**	-10.8414	0.006	-18.5008	-3.18193
**Marital Status(Partner)**	14.3645	0.005	4.408108	24.3209
**Marital Status (Previous Partner)**	12.06564	0.054	-0.22011	24.35138
**Baseline Anxiety (Anxious)**	0.638665	0.888	-8.27675	9.554082
**Baseline Depression (Depressed)**	-16.5488	0.005	-28.1469	-4.95066
**Cardiac Treatment (PCI)**	-10.3835	0.106	-22.956	2.188905
**Cardiac Treatment (CABG)**	3.094895	0.656	-10.5353	16.72513
**Cardiac Treatment (Other)**	-10.3174	0.125	-23.4846	2.849781
**Age (years)**	-3.49547	<0.001	-3.85631	-3.13462
**Total Comorbidities**	-3.81197	<0.001	-5.49696	-2.12698
**Mode of Delivery (Self-delivered)**	0.31067	0.957	-11.111	11.73234
**Staff Hours (hours)**	0.036993	0.177	-0.01673	0.090718
**MDT Team (>=3 staff types)**	-9.03675	0.264	-24.8901	6.81657
**Volume (number of patients)**	-0.00672	0.606	-0.03228	0.018839
**Constant**	435.464	<0.001	400.3336	470.5945
R^2 = 0.69, included sample size 6,175				
